# Gender differences in pre-surgical motivations, expectations, and patient-reported outcomes in Class III orthognathic surgery patients: An item-level analysis

**DOI:** 10.1371/journal.pone.0362253

**Published:** 2026-11-05

**Authors:** Gizem Altundal, Asuman Deniz Gümrü Celikel, Gülnaz Marsan

**Affiliations:** 1 Department of Orthodontics, Faculty of Dentistry, Istanbul Kent University, Istanbul, Turkey; 2 Department of Orthodontics, Faculty of Dentistry, Istanbul University, Istanbul, Turkey; Universidade Federal Fluminense, BRAZIL

## Abstract

**Objective:**

To evaluate gender differences in pre-surgical motivations, expectations, functional and psychosocial evaluations, and treatment process perceptions among adult patients with skeletal Class III malocclusion undergoing orthodontic preparation for orthognathic surgery.

**Methods:**

In this single-center, cross-sectional study, 81 adults (37 females, 44 males) with skeletal Class III malocclusion completed a 27-item investigator-developed questionnaire during pre-surgical orthodontic preparation, covering demographics, problem awareness, functional and psychosocial evaluations, motivation and expectations, treatment process, and patient engagement. Gender differences were analyzed item by item with Mann-Whitney U, chi-square, or Fisher’s exact tests. P-values for the 37 comparisons were adjusted with the Benjamini-Hochberg false discovery rate (FDR) procedure within questionnaire domains, and effect sizes with 95% confidence intervals were estimated. Because the a priori sample size calculation did not address between-gender comparisons, these were treated as exploratory.

**Results:**

Seven differences met the unadjusted threshold (p < 0.05), of which three remained significant after within-domain FDR correction: females reported greater lack of self-confidence in social environments (q = 0.044), and males reported more adequate information from their orthodontist (q = 0.018) and higher pre-operative treatment satisfaction (q = 0.019), with moderate effect sizes (rank-biserial |r| = 0.30–0.35). The other four, all reported more often by females (eating difficulty, food avoidance, temporomandibular joint problems, and lack of confidence when photographed), were nominally significant only (unadjusted p = 0.013–0.043). No difference survived correction across all 37 comparisons. For the other items no significant differences were detected, but confidence intervals did not exclude small-to-moderate differences.

**Conclusions:**

Gender differences in social self-confidence, perceived information adequacy, and pre-operative satisfaction were the strongest exploratory findings. Because the study was not powered for gender comparisons, non-significant results do not indicate that the genders are similar. These exploratory findings warrant confirmation in adequately powered multi-center, longitudinal studies.

## Introduction

Patients with dentofacial deformities require treatment due to low self-perception, decreased confidence levels, and physiological problems related to the deformity [1]. In addition to affecting oral health and function, these deformities also negatively affect mental health and reduce quality of life [1]. Although difficulty and discomfort in chewing, temporomandibular joint pain, and functional problems are important reasons for requesting treatment, facial appearance during social interactions and the resulting feelings of inadequacy have been reported to be equally or more important [1–3]. For this reason, the evaluation of patients’ thoughts and expectations alongside clinical examination and diagnostic records is emphasized as a means of improving treatment outcomes and quality of life [2]. Orthognathic surgery is therefore regarded as a psychological intervention in addition to a physical one, as there is evidence that it provides psychological benefits including increased self-esteem, social adaptation, and improved quality of life [4,5].

Over the years, much of the research has focused on improving the aesthetics of patients’ facial and dental components [3,6,7]. Rivera et al. reported that 71.3% of patients cited esthetic improvements as their primary motivation [3], and Williams et al. found that 80% of patients sought to straighten their teeth and 63% wanted to improve their looks [7]. Because orthognathic surgery directly alters the patient’s appearance within a short period, measuring its psychosocial impact is as important as assessing clinical outcomes. Individuals’ motivations, perceptions, and expectations play an important role in determining not only surgical success but also psychosocial success [1]. Although satisfaction with orthognathic treatment is generally high, some patients may be dissatisfied with functional recovery results, and this dissatisfaction is related to how the patient is affected by the problem as well as to personality, motivation, and expectations [8–10].

Patients with Class III malocclusion typically present with an affected facial profile, dental misalignment, and impairments in mastication and speech that can substantially affect quality of life [11,12]. In a nationwide Turkish meta-analysis, the pooled prevalence of Class III malocclusion in the general population was approximately 11% [13]. However, among Turkish adults referred for orthognathic surgery, Olkun et al. reported that Class III malocclusion was the most prevalent indication, accounting for 69% of cases [11].

A growing body of evidence indicates that pre-surgical motivations, expectations, and quality of life among orthognathic patients are not uniformly distributed between genders. Williams et al. reported that female patients were significantly more self-conscious about their facial appearance and teeth and more likely to avoid smiling in photographs than male patients [7]. Rivera et al. similarly found that female patients were more likely than males to cite temporomandibular joint–related concerns as a reason for seeking orthognathic surgery [3]. Bortoluzzi et al. found that Brazilian female patients scored significantly higher (indicating worse quality of life) than males across multiple OQLQ domains, including facial esthetics, oral function, and awareness of facial deformity [14]. Johansson et al., in a Swedish national register cohort, similarly reported that women graded facial esthetics, oral function, and awareness of facial deformity significantly higher than men before surgery [15]. These findings collectively suggest that gender is a factor shaping the pre-surgical experience of orthognathic patients.

Despite this accumulating evidence, important gaps remain. Most prior studies have assessed gender differences at the domain level of the OQLQ, which combines multiple items into summary scores and can mask item-specific differences or produce null findings when individual items within a domain diverge [12,15]. The aim of this study was to evaluate the pre-surgical motivations, expectations, functional and psychosocial evaluations, and treatment process perceptions of orthognathic surgery patients, with particular attention to gender differences at the item level among Turkish adult patients with Class III skeletal anomalies.

## Materials and methods

### Study design and setting

This single-center, cross-sectional study was conducted at the Department of Orthodontics, Faculty of Dentistry, Istanbul University. The study protocol was approved by the Clinical Research Ethics Committee of Istanbul University Faculty of Dentistry (decision no. 587, meeting no. 119, protocol no. 2022/16; dated 8 March 2022). Participants were recruited between 1 April 2022 and 1 October 2022. All participants provided written informed consent prior to enrollment. The study is reported in accordance with the STROBE statement for cross-sectional studies (S1 File).

### Participants

Patients aged 18 years and older undergoing pre-surgical orthodontic preparation for orthognathic surgery to correct single or double jaw deformities were assessed for eligibility. Inclusion required a diagnosis of skeletal Class III malocclusion. Exclusion criteria were: age under 18 years, cleft lip and/or palate, congenital deformities affecting dentofacial development, and prior orthodontic and/or orthognathic surgical treatment.

Of 105 patients assessed for eligibility, 20 were not eligible (10 with cleft lip and/or palate, 2 with other syndromic craniofacial conditions, and 8 with skeletal Class II malocclusion). All 85 eligible patients agreed to participate; none declined. Four questionnaires were excluded because they were incomplete or incorrectly completed, in accordance with the study protocol, leaving 81 participants for analysis (S1 Fig). Although the approved study protocol encompassed patients with anteroposterior and/or vertical skeletal discrepancies, enrollment was restricted to patients with skeletal Class III malocclusion because this was the predominant diagnostic category presenting for orthognathic preparation during the recruitment period and a diagnostically homogeneous cohort was considered methodologically preferable. This restriction was applied during eligibility assessment, before questionnaire administration; therefore, patients with skeletal Class II malocclusion were not enrolled. Eligibility and exclusion counts were determined retrospectively from clinical records.

### Sample size determination

An a priori power analysis was carried out in February 2022 by an external statistician, before ethics committee submission and participant recruitment, and is documented in the approved study protocol. Using G*Power 3.1, the calculation was specified as a one-sample t-test (difference from a constant), one-tailed, with an anticipated effect size of d = 0.37, α = 0.05, and power = 0.95. This indicated a required total sample of 81 participants (actual power = 0.951; noncentrality parameter δ = 3.33, critical t = 1.664, df = 80). The statistician’s report cites four prior orthognathic surgery questionnaire studies as the literature considered in planning [3,7,16,17], but it does not document how the value of d = 0.37 was derived from them, does not give a reason for the one-tailed specification, and does not name a primary questionnaire outcome or a numerical reference value for the test. No further contemporaneous documentation of these choices is available. In accordance with the protocol, data collection was terminated once this pre-specified number of complete questionnaires had been obtained; the final sample comprised 81 participants (37 females, 44 males).

The statistician’s report states that a one-sample test was chosen because the study was planned as a single-group study. The calculation therefore applies to the sample as a whole and does not provide a sample size justification for the between-gender comparisons reported here. Those comparisons were not powered a priori and are presented as exploratory. To make their sensitivity explicit, power was calculated for the realized group sizes (37 females, 44 males; two-sided α = 0.05). The power to detect the originally assumed effect of d = 0.37 was 0.37, and the smallest effect detectable with 80% power was d ≈ 0.63 (d ≈ 0.65 for the Mann-Whitney U test, assuming an asymptotic relative efficiency of 0.955). Detecting d = 0.37 with 80% power would have required approximately 116 participants per group. The study could therefore detect only moderate-to-large gender differences, and non-significant results are interpreted in the light of their confidence intervals rather than as evidence of no difference (see Significance Threshold and Multiplicity).

### Survey instrument

A structured questionnaire comprising 27 items was administered to all participants during pre-surgical orthodontic preparation (S1 Table). The instrument was developed by the investigators for this study rather than adopted from a single validated scale. As documented in the approved study protocol, items were prepared by reviewing previously published orthognathic surgery questionnaires [3,7,16,17] and by writing additional items to capture the local clinical context. The instrument covers constructs that overlap substantially with those of the Orthognathic Quality of Life Questionnaire (OQLQ) [18,19], namely oral function, facial aesthetics, social aspects of deformity, and awareness of facial deformity. Items taken from these sources were reworded for the Turkish clinical setting and, where the original used a binary or bother-based response, were converted to the 5-point frequency format used throughout the instrument. Additional items covering breathing during the day and during sleep, perceived effect of appearance on success in life, and monthly income were written by the investigators to capture the local clinical context. The full instrument as administered is provided in S1 Table.

Items were organized into seven domains: demographic information (Q1–Q4), problem awareness (Q5–Q6), functional evaluations (Q7–Q12), psychosocial evaluations (Q13–Q19), motivation and treatment expectations (Q20–Q21), treatment process evaluation (Q22–Q24), and patient engagement (Q25–Q27). Functional, psychosocial, and treatment process items used a 5-point Likert scale of response frequency (1 = Never to 5 = Always). Motivation source (Q20) and treatment expectations (Q21) used single- and multiple-choice formats, respectively. Patient engagement items (Q25–Q26) included conditional follow-up questions administered only to participants who responded affirmatively.

Because the response format was framed in terms of symptom or experience frequency rather than the degree of bother used by the OQLQ, and because items were drawn from several sources, the resulting instrument is not equivalent to any previously validated scale. The Turkish version of the OQLQ (OQLQ-TR), for which cross-cultural adaptation and psychometric testing have since been reported [20], had not been published when this study was designed, and its validation does not extend to the instrument used here. The Turkish wording of items derived from English-language sources was prepared by the investigators; items originating from Türker et al. [16], a study conducted in Turkish patients, retained their original Turkish phrasing. Internal consistency of the Likert-scale domains in the present sample is reported in the Results. No formal cross-cultural validation, test-retest reliability assessment, or construct validity analysis was undertaken, and this is acknowledged as a limitation.

All questionnaires were self-completed in the clinic during routine orthodontic appointments, with the investigator available to clarify items on request. No time limit was imposed, and no questionnaires were administered by post. Participation had no bearing on the patient’s treatment.

### Statistical analysis and data visualization

All statistical analyses were performed using Python (version 3.13.6) with SciPy (1.16.3), pandas (2.3.3), NumPy (2.3.3), and statsmodels (0.14.6). Data visualization was performed using matplotlib (3.10.8) and seaborn (0.13.2).

For all Likert-scale items, descriptive percentages are reported throughout as the combined “Often/Always” categories, with the combined “Never/Rarely” categories reported where the contrast is informative. This convention is applied consistently across the Results and Discussion.

We followed the analytical approach used in prior studies employing similar survey instruments in orthognathic surgery populations [3,7,16,17]. The choice of statistical test for each question was determined by the measurement level of the dependent variable:

*Continuous data (Q2, age).* Normality was assessed using the Shapiro-Wilk test for each gender group. As at least one group deviated from normality, the Mann-Whitney U test was applied for between-group comparison.

*Ordinal data (Q3–Q4, Q7–Q19, Q22–Q24).* The Mann-Whitney U test was used as the primary test, as it respects the ordinal nature of Likert-type responses and does not assume normality. For the demographic ordinal questions (Q3–Q4), chi-square tests of independence are additionally reported as supplementary analyses.

*Nominal categorical data (Q5, Q6, Q20).* Chi-square tests of independence were used to evaluate the association between gender and response categories. Where expected cell counts fell below 5, Monte Carlo exact tests were performed and are reported alongside the chi-square result.

*Multiple-choice data (Q21).* Each of the 10 expectation categories was analyzed independently as a binary outcome (selected/not selected). Chi-square tests of independence or Fisher’s exact tests (when expected cell counts were insufficient) were used for each category.

*Binary and categorical data (Q25–Q27).* Chi-square tests of independence were used for gender comparisons, with Fisher’s or Monte Carlo exact tests applied when cell counts warranted. Conditional questions (Q25.1, Q26.1) were analyzed only among the subset of participants who answered “Yes” to the corresponding parent question.

*Internal consistency.* Cronbach’s alpha was calculated for each of the three Likert-scale domains, with bias-corrected bootstrap confidence intervals based on 2,000 resamples.

### Effect size measures and confidence intervals

Wilson Score confidence intervals (95%) were calculated for all reported proportions, following Wilson [21]; this method was selected for its superior coverage properties with small to moderate sample sizes compared with the Wald interval. Effect sizes were calculated for every comparison, with 95% confidence intervals where applicable, so that the magnitude and precision of each estimate, including non-significant ones, could be assessed. For Mann-Whitney U tests, the rank-biserial correlation r (equivalent to Cliff’s delta) was calculated, with positive values indicating higher scores among females and negative values higher scores among males; its confidence interval was obtained with Cliff’s asymmetric method [22]. Absolute values of about 0.1, 0.3, and 0.5 were taken to indicate small, moderate, and large effects. For binary items, the difference in proportions (females minus males, in percentage points) was calculated with a Newcombe hybrid score confidence interval [23]. For nominal items with more than two categories, Cramér’s V was reported as a descriptive effect-size measure without confidence intervals.

### Significance threshold and multiplicity

In total, 37 between-gender comparisons were performed across the 27 items and their sub-components. To control the false discovery rate, p-values were adjusted with the Benjamini-Hochberg procedure [24] within each questionnaire domain (demographics, problem awareness, functional evaluations, psychosocial evaluations, motivation and treatment expectations, treatment process evaluation, and patient engagement; 3, 2, 6, 7, 11, 3, and 5 comparisons, respectively). The domains reflect the content grouping of the questionnaire items used to present the results (Figs 1–9) and were not formed on the basis of the gender-comparison results. The use of Benjamini-Hochberg correction within these domains was not specified in the 2022 study protocol; it was introduced during peer review. Adjusted values (q) are reported alongside unadjusted p-values in Tables 1–3. A difference was considered statistically significant if q < 0.05; differences with unadjusted p < 0.05 but q ≥ 0.05 are described as nominally significant and are treated as hypothesis-generating. As a more conservative check, the same procedure was applied across all 37 comparisons simultaneously (S2 Table). Results that were not statistically significant are reported as showing no statistically significant difference and were not interpreted as evidence of equivalence; their effect-size confidence intervals are reported to show the range of differences compatible with the data.

**Fig 1:**
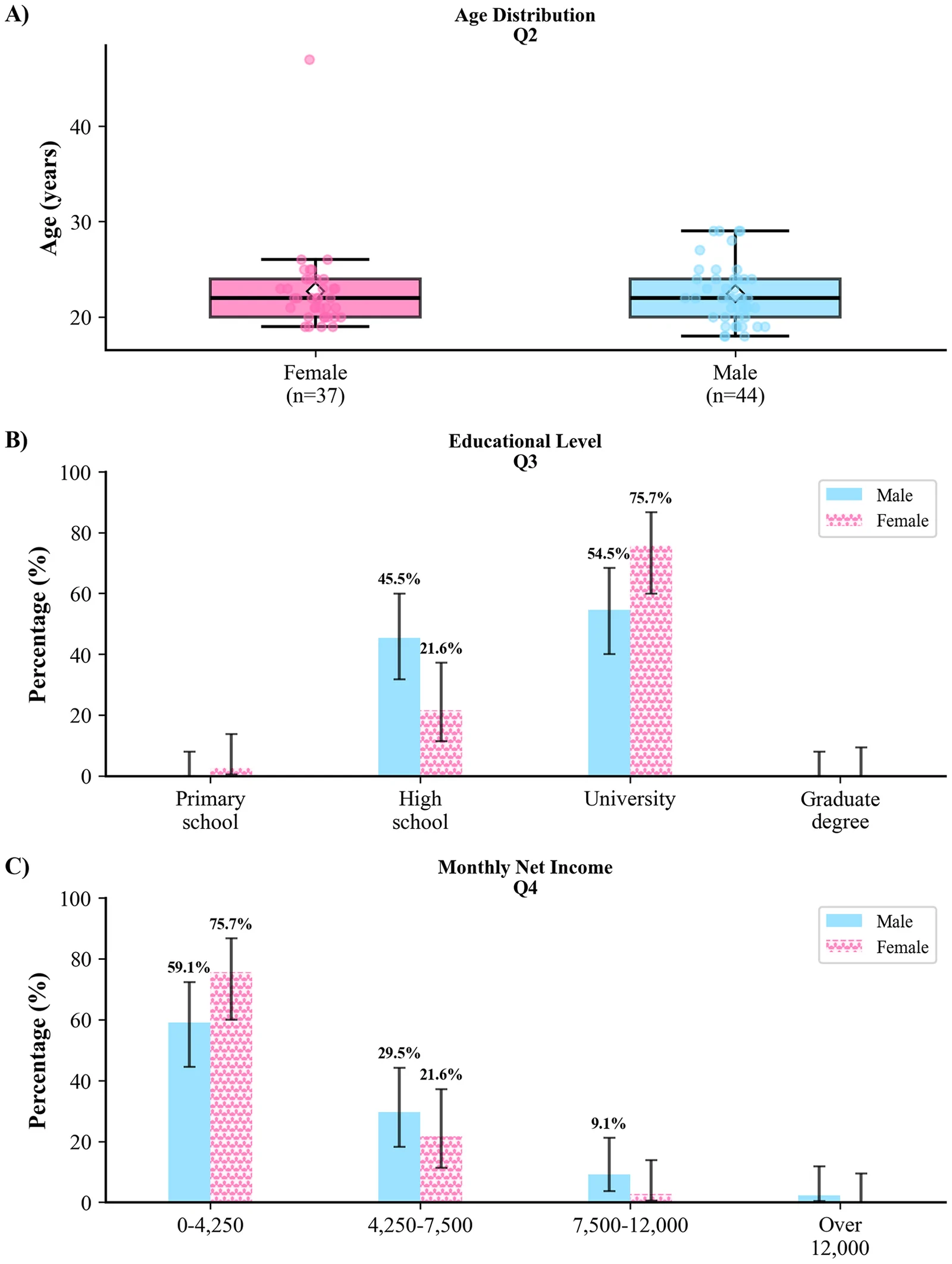
Demographic characteristics of the study population by gender. (A) Age distribution for female (n = 37) and male (n = 44) participants. Box plots show median, interquartile range, and individual data points; diamond markers indicate the mean. (B) Distribution of educational level by gender (Q3). (C) Distribution of monthly net income in Turkish Lira by gender (Q4). All p-values are from Mann–Whitney U tests. Error bars represent 95% Wilson Score confidence intervals.

**Fig 2:**
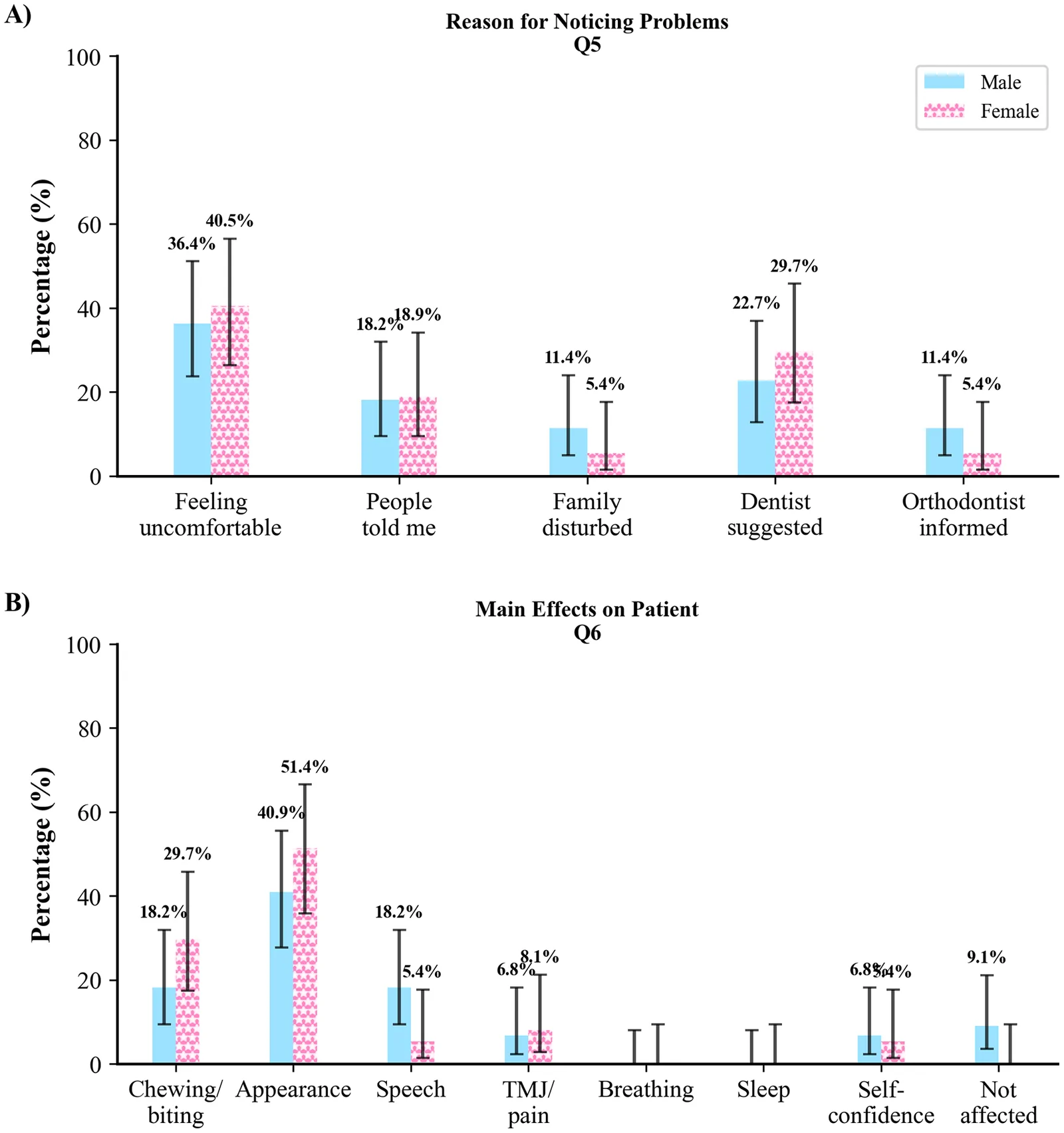
Problem awareness and perceived impact by gender. (A) Distribution of reasons for noticing dentofacial problems (Q5). (B) Distribution of the main reported effects of the condition on participants (Q6). Chi-square tests were used for both comparisons. Error bars represent 95% Wilson Score confidence intervals.

**Fig 3:**
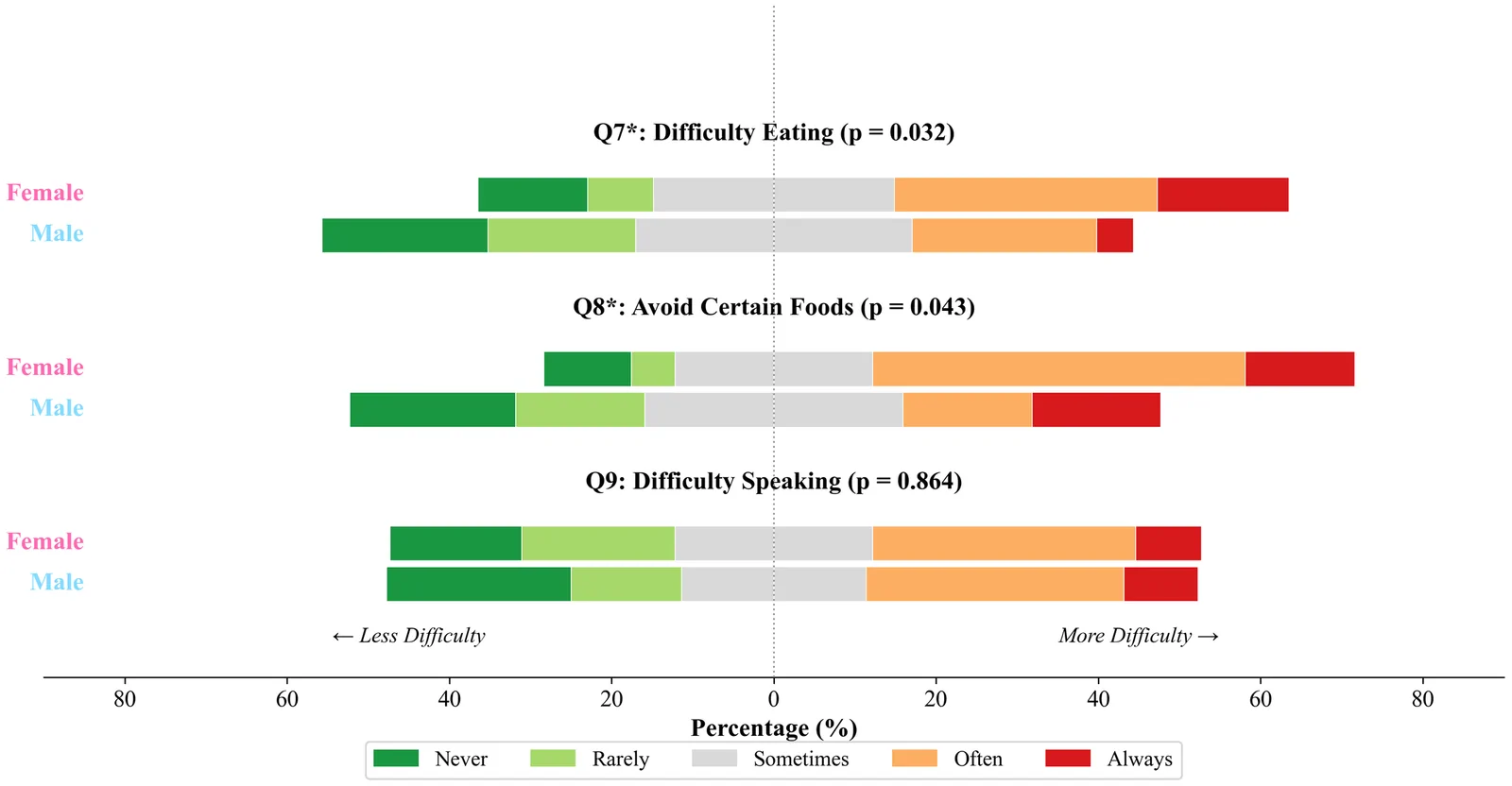
Functional evaluations of eating and speech difficulties by gender. Diverging stacked bar charts show the distribution of Likert-scale responses (1 = Never to 5 = Always) for Q7, Q8, and Q9. Negative values represent lower difficulty (Never, Rarely), and positive values represent higher difficulty (Often, Always), with Sometimes centered at zero. All p-values are from Mann–Whitney U tests. Female, n = 37; Male, n = 44. *Unadjusted p < 0.05; FDR-adjusted values are given in Tables 2 and 3.

**Fig 4:**
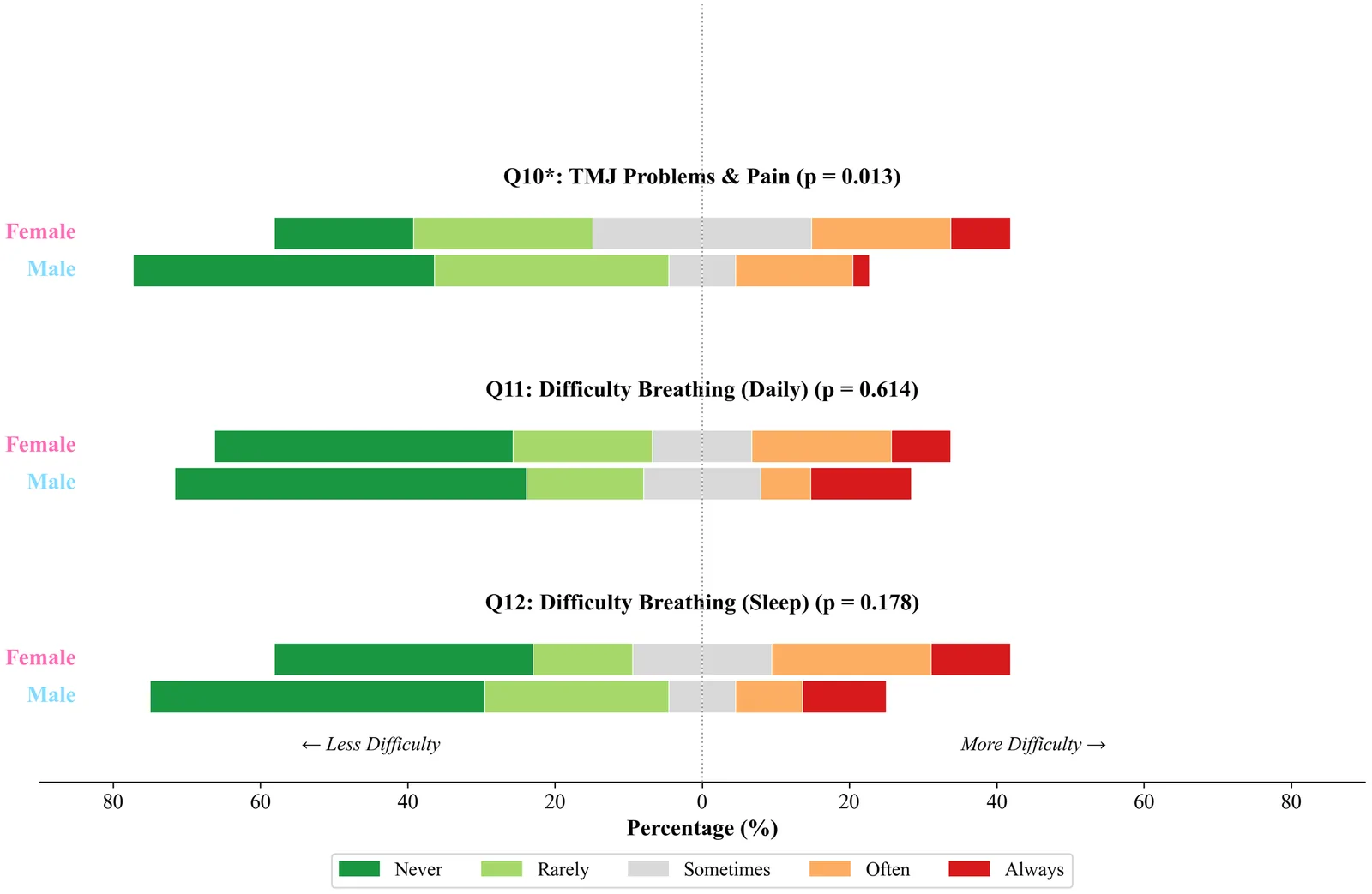
Functional evaluations of temporomandibular joint problems and breathing difficulties by gender. Diverging stacked bar charts show the distribution of Likert-scale responses (1 = Never to 5 = Always) for Q10, Q11, and Q12. Negative values represent lower difficulty (Never, Rarely), and positive values represent higher difficulty (Often, Always), with Sometimes centered at zero. All p-values are from Mann–Whitney U tests. Female, n = 37; Male, n = 44. *Unadjusted p < 0.05; FDR-adjusted values are given in Tables 2 and 3.

**Fig 5:**
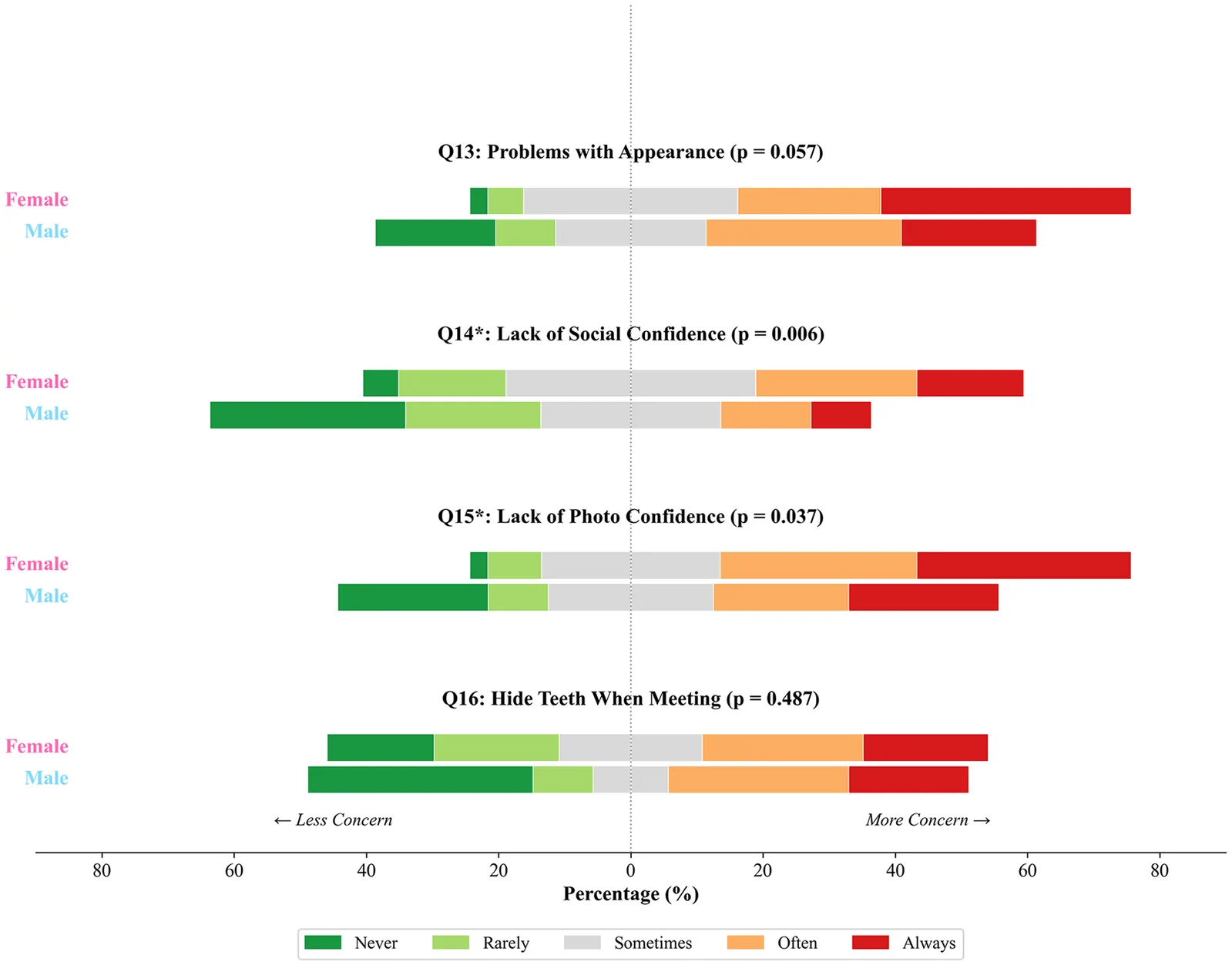
Psychosocial evaluations of appearance concerns and self-confidence by gender. Diverging stacked bar charts show the distribution of Likert-scale responses (1 = Never to 5 = Always) for Q13, Q14, Q15, and Q16. Negative values represent less concern (Never, Rarely), and positive values represent more concern (Often, Always), with Sometimes centered at zero. All p-values are from Mann–Whitney U tests. Female, n = 37; Male, n = 44. *Unadjusted p < 0.05; FDR-adjusted values are given in Tables 2 and 3.

**Fig 6:**
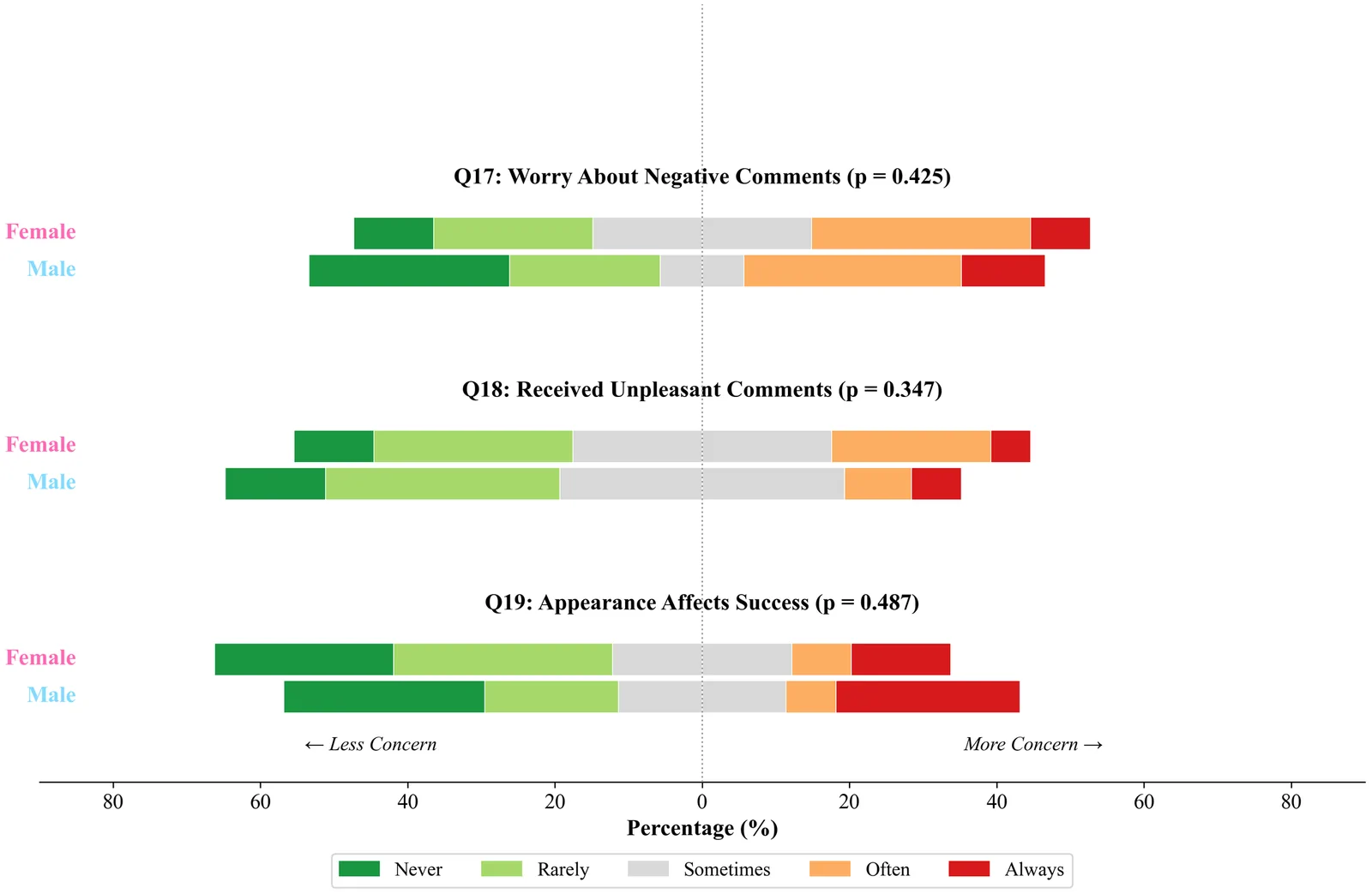
Psychosocial evaluations of social impact by gender. Diverging stacked bar charts show the distribution of Likert-scale responses (1 = Never to 5 = Always) for Q17, Q18, and Q19. Negative values represent lower-frequency responses (Never, Rarely), and positive values represent higher-frequency responses (Often, Always), with Sometimes centered at zero. All p-values are from Mann–Whitney U tests. Female, n = 37; Male, n = 44.

**Fig 7:**
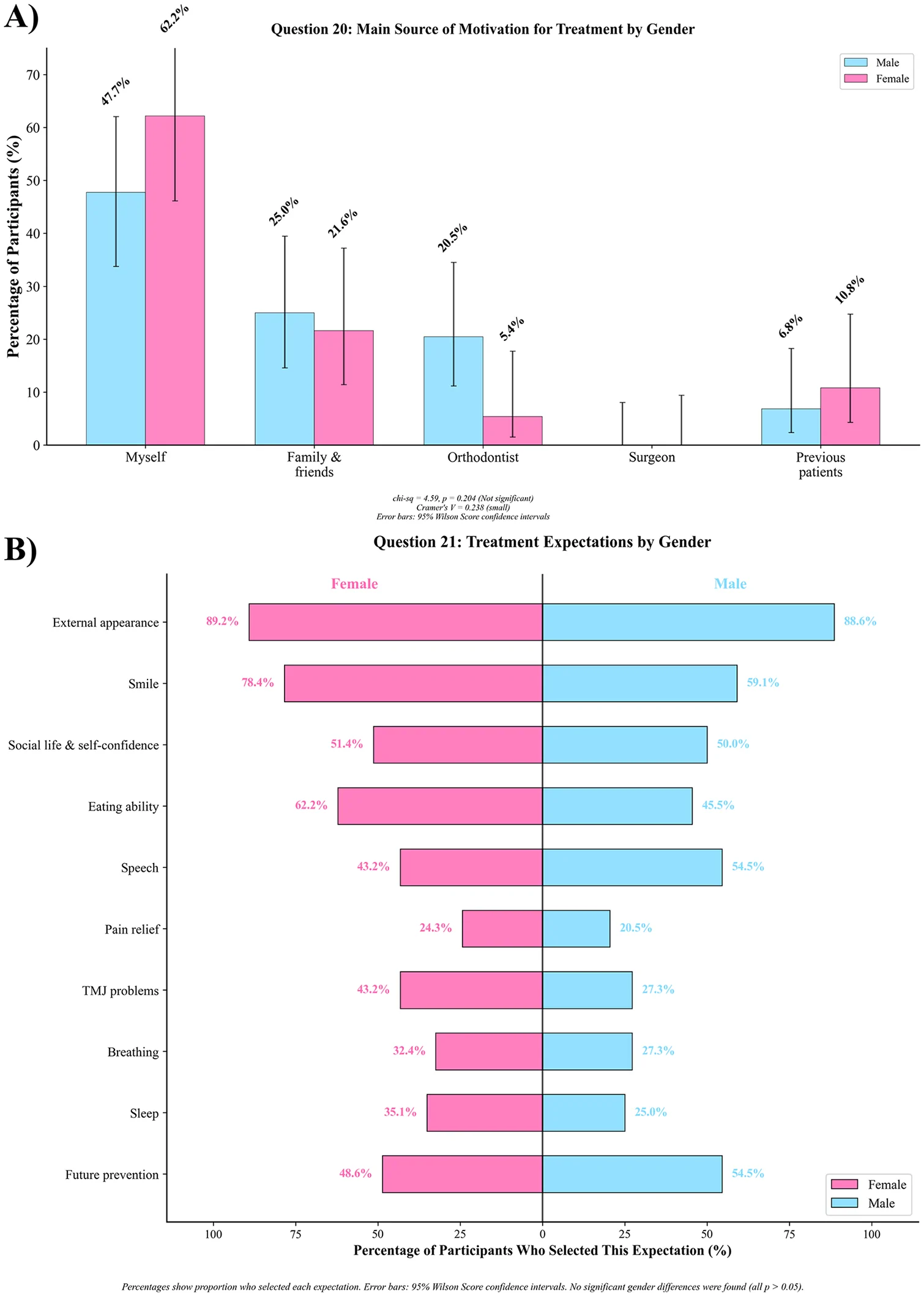
Treatment motivation and expectations by gender. (A) Distribution of primary motivation sources for treatment (Q20); a chi-square test was used for the gender comparison. (B) Bipolar diverging bar chart showing the percentage of participants selecting each treatment expectation category (Q21.1–Q21.10). No statistically significant gender differences were detected across the expectation categories (all p > 0.05). Error bars represent 95% Wilson Score confidence intervals. Female, n = 37; Male, n = 44.

**Fig 8:**
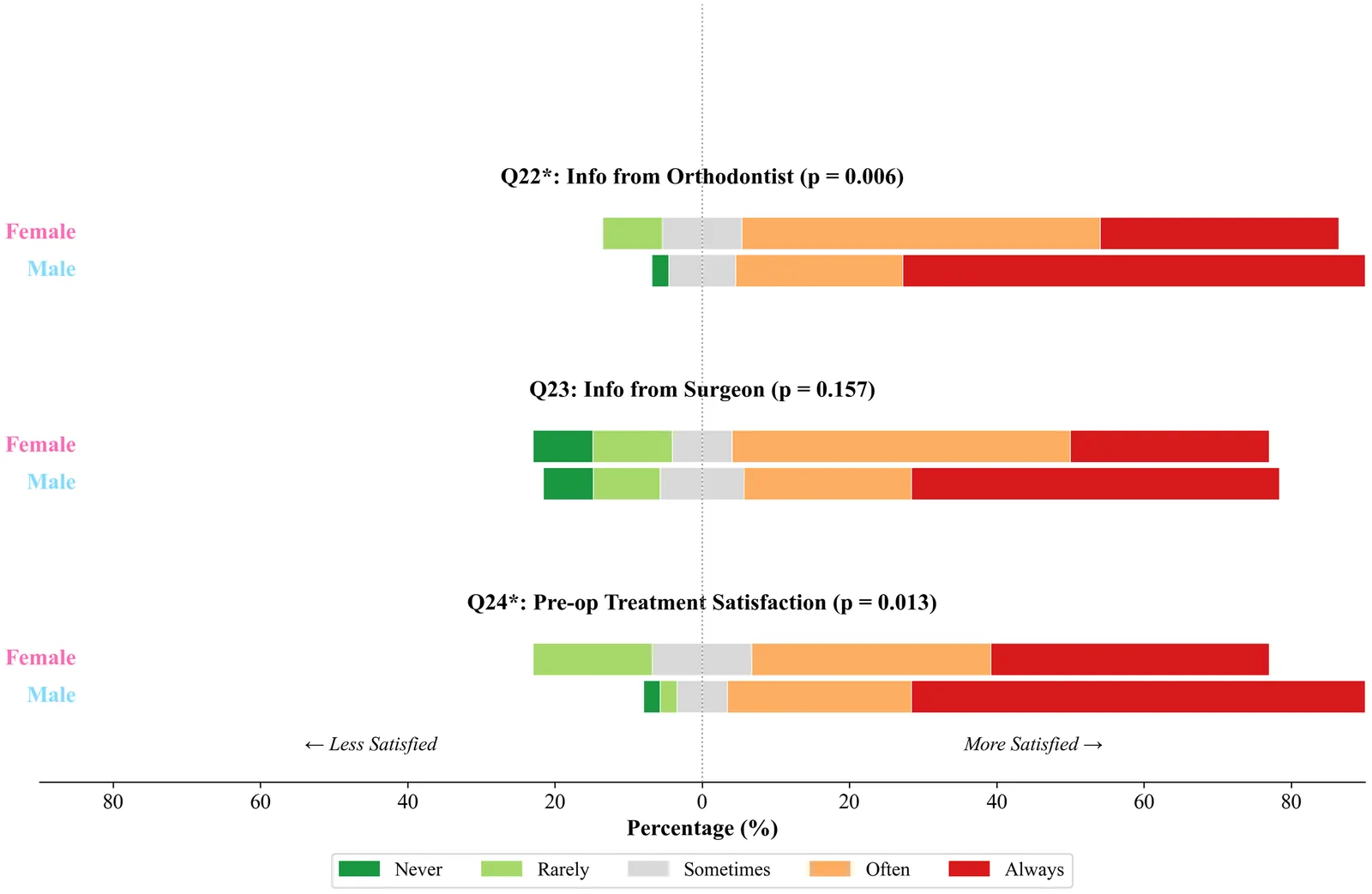
Treatment process evaluation by gender. Diverging stacked bar charts show the distribution of Likert-scale responses (1 = Never to 5 = Always) for Q22, Q23, and Q24. Negative values represent less satisfaction (Never, Rarely), and positive values represent more satisfaction (Often, Always), with Sometimes centered at zero. All p-values are from Mann–Whitney U tests. Female, n = 37; Male, n = 44. *Unadjusted p < 0.05; FDR-adjusted values are given in Tables 2 and 3.

**Fig 9:**
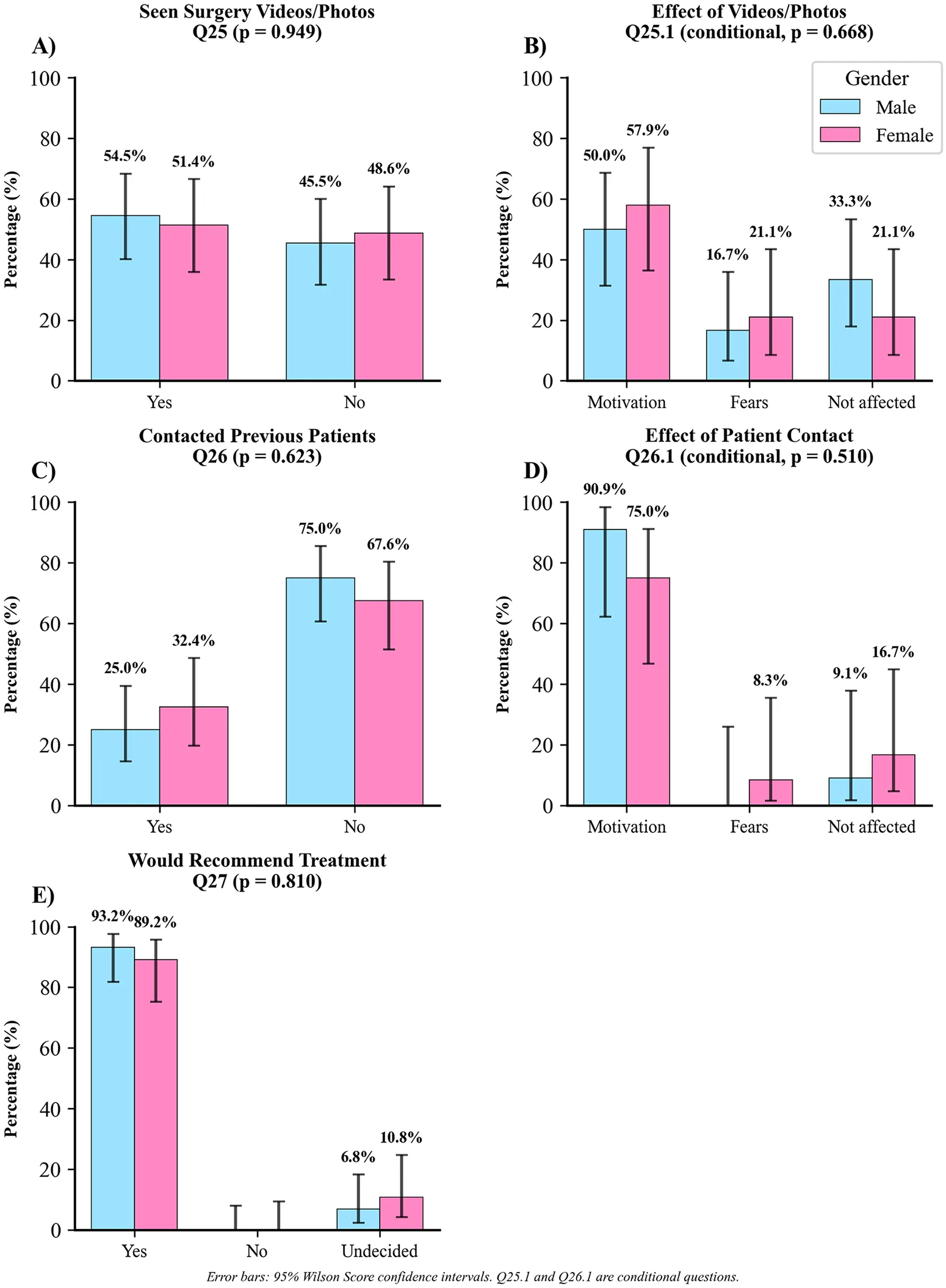
Patient engagement behaviors and treatment recommendation by gender. (A) Exposure to surgery-related videos and photos (Q25). (B) Perceived effect of viewing such materials among those who reported exposure (Q25.1). (C) Contact with previous surgery patients (Q26). (D) Perceived effect of patient contact among those who reported contact (Q26.1). (E) Willingness to recommend treatment to others (Q27). Chi-square tests were used for all comparisons. Error bars represent 95% Wilson Score confidence intervals. Female, n = 37; Male, n = 44.

## Results

### Baseline characteristics

A total of 81 patients were included in this study. The study population consisted of 37 females (45.7%) and 44 males (54.3%). The overall mean age was 22.6 ± 3.8 years, ranging from 18 to 47 years.

Internal consistency was acceptable across all three Likert-scale domains: functional evaluations (Q7–Q12), α = 0.77 (95% CI 0.66–0.83); psychosocial evaluations (Q13–Q19), α = 0.91 (95% CI 0.87–0.93); and treatment process evaluation (Q22–Q24), α = 0.71 (95% CI 0.58–0.81). All item-rest correlations were positive and exceeded 0.37.

Detailed statistical comparisons for all survey questions are provided in Tables 1–3, organized by domain: patient characteristics and problem awareness (Table 1), functional and psychosocial assessments (Table 2), and treatment process evaluations (Table 3). For every comparison the tables give the unadjusted p-value, the within-domain FDR-adjusted q-value, and the effect size with its 95% confidence interval where applicable.

**Table 1:** Patient characteristics and problem awareness: Gender comparison (females vs males).

Question	Description	p	q	Effect size (95% CI)	Figure
***Demographics***					
Q2	Age	0.973	0.973	r = 0.00 (−0.25 to 0.24)	Fig 1
Q3	Educational level	0.066 (0.053)	0.134	r = 0.20 (−0.01 to 0.39)	
Q4	Monthly net income	0.089 (0.323)	0.134	r = −0.18 (−0.37 to 0.02)	
***Problem awareness***					
Q5	What caused you to notice these problems?	0.712	0.712	V = 0.16	Fig 2
Q6	How does your current condition mainly affect you?	0.170	0.341	V = 0.31	

Q2–Q4: p-values from Mann-Whitney U tests; supplementary chi-square results are given in parentheses. Q5–Q6: p-values from chi-square tests of independence. For Q3 and Q4 some expected cell counts were below 5; Monte Carlo exact tests gave p = 0.034 and p = 0.297 respectively. q: Benjamini-Hochberg false discovery rate-adjusted p-value within domain. r: rank-biserial correlation with 95% confidence interval (positive values indicate higher scores among females). V: Cramér’s V. *Unadjusted p < 0.05.

**Table 2:** Functional and psychosocial impact assessment: Gender comparison (females vs males).

Question	Description	p	q	Effect size (95% CI)	Figure
***Functional evaluations — eating and speech***					
Q7	Do you have difficulty eating?	0.032*	0.085	r = 0.27 (0.02 to 0.49)	Fig 3
Q8	Do you avoid eating certain foods due to problems with your teeth and jaw?	0.043*	0.085	r = 0.26 (0.01 to 0.48)	
Q9	Do you have difficulty speaking and pronouncing words?	0.864	0.864	r = 0.02 (−0.22 to 0.26)	
***Functional evaluations — TMJ and breathing***					
Q10	Does your current condition cause temporomandibular joint problems and pain?	0.013*	0.076	r = 0.31 (0.07 to 0.52)	Fig 4
Q11	Do you have difficulty breathing in daily life?	0.614	0.737	r = 0.06 (−0.18 to 0.30)	
Q12	Do you have difficulty breathing while sleeping?	0.178	0.267	r = 0.17 (−0.08 to 0.40)	
***Psychosocial evaluations — appearance and confidence***					
Q13	Do you have problems with your external appearance?	0.057	0.133	r = 0.24 (−0.01 to 0.46)	Fig 5
Q14	Do you feel a lack of self-confidence when entering any social environment?	0.006*	0.044†	r = 0.35 (0.10 to 0.55)	
Q15	Do you feel a lack of self-confidence when being photographed?	0.037*	0.129	r = 0.26 (0.02 to 0.48)	
Q16	Do you hide your teeth when you meet people?	0.487	0.487	r = 0.09 (−0.16 to 0.32)	
***Psychosocial evaluations — social impact***					
Q17	Are you worried about receiving negative comments about your appearance?	0.425	0.487	r = 0.10 (−0.15 to 0.34)	Fig 6
Q18	Have you ever received unpleasant comments about your appearance?	0.347	0.487	r = 0.12 (−0.13 to 0.35)	
Q19	Do you think your appearance affects your life success?	0.487	0.487	r = −0.09 (−0.32 to 0.16)	

All p-values from Mann-Whitney U tests. q: Benjamini-Hochberg false discovery rate-adjusted p-value within domain. r: rank-biserial correlation with 95% confidence interval (positive values indicate higher scores among females). Female, n = 37; male, n = 44. *Unadjusted p < 0.05; †q < 0.05.

**Table 3:** Treatment process and patient engagement: gender comparison (females vs males).

Question	Description	p	q	Effect size (95% CI)	Figure
***Motivation***					
Q20	What is your main source of motivation for this treatment?	0.204	0.562	V = 0.24	Fig 7
***Treatment expectations***					
Q21.1	Expectation: external appearance	1.000	1.000	Δ = 0.6 (−14.8 to 14.8)	Fig 7
Q21.2	Expectation: smile	0.107	0.562	Δ = 19.3 (−1.1 to 37.2)	
Q21.3	Expectation: social life and self-confidence	1.000	1.000	Δ = 1.4 (−19.6 to 22.1)	
Q21.4	Expectation: eating ability	0.201	0.562	Δ = 16.7 (−4.9 to 36.2)	
Q21.5	Expectation: speech	0.429	0.830	Δ = −11.3 (−31.3 to 10.2)	
Q21.6	Expectation: pain relief	0.882	1.000	Δ = 3.9 (−13.9 to 22.2)	
Q21.7	Expectation: TMJ problems	0.204	0.562	Δ = 16.0 (−4.6 to 35.2)	
Q21.8	Expectation: ease in breathing	0.793	1.000	Δ = 5.2 (−14.2 to 24.6)	
Q21.9	Expectation: comfortable sleep	0.453	0.830	Δ = 10.1 (−9.5 to 29.3)	
Q21.10	Expectation: prevention of future problems	0.760	1.000	Δ = −5.9 (−26.4 to 15.3)	
***Treatment process evaluation***					
Q22	Have you received sufficient information about the treatment from your orthodontist?	0.006*	0.018†	r = −0.33 (−0.52 to −0.09)	Fig 8
Q23	Have you received sufficient information about the treatment from your surgeon?	0.157	0.157	r = −0.17 (−0.40 to 0.07)	
Q24	Are you satisfied with your pre-operative orthodontic treatment process?	0.013*	0.019†	r = −0.30 (−0.50 to −0.06)	
***Patient engagement***					
Q25	Have you seen videos and photos related to before and after surgery?	0.949	0.949	Δ = −3.2 (−23.9 to 17.8)	Fig 9
Q25.1	How did these recordings affect you?	0.668	0.949	V = 0.14	
Q26	Have you contacted patients who have previously had surgery?	0.623	0.949	Δ = 7.4 (−11.9 to 26.6)	
Q26.1	How did this contact affect you?	0.510	0.949	V = 0.24	
Q27	Would you recommend this treatment to other patients in your condition?	0.810	0.949	Δ = −4.0 (−18.6 to 9.1)	

Q20, Q21.1–Q21.10 and Q25–Q27: p-values from chi-square tests of independence, or Fisher’s or Monte Carlo exact tests where expected cell counts were below 5. Q22–Q24: Mann-Whitney U tests. Conditional items were analyzed only among participants answering affirmatively to the parent question (Q25.1, n = 43; Q26.1, n = 23); for Q26.1 the expected cell counts were small and the Monte Carlo exact test gave p = 1.000. q: Benjamini-Hochberg false discovery rate-adjusted p-value within domain. r: rank-biserial correlation with 95% confidence interval (positive values indicate higher scores among females). Δ: difference in the proportion answering “selected” or “yes” (females minus males, percentage points) with 95% Newcombe confidence interval. V: Cramér’s V. Female, n = 37; male, n = 44. *Unadjusted p < 0.05; †q < 0.05.

Of the 37 comparisons, seven reached nominal significance (unadjusted p < 0.05) and three remained significant after within-domain FDR correction (Q14, Q22, and Q24). For the ordinal outcome items without a statistically significant difference, the 95% confidence intervals of the rank-biserial correlation extended to absolute values between 0.26 and 0.46, and for the binary items the confidence intervals for the difference in proportions extended to between 15 and 37 percentage points. Differences of small-to-moderate size therefore cannot be excluded for these items.

### Demographic characteristics by gender (Questions 2–4)

Fig 1 and Table 1 present patient characteristics including age distribution, educational level, and monthly income.

In Fig 1A (Question 2), female participants demonstrated a mean age of 22.7 ± 4.6 years, and male participants a mean age of 22.5 ± 3.1 years. No statistically significant age difference was detected between genders (p = 0.973).

In Fig 1B (Question 3), both male and female participants demonstrated a median educational level corresponding to university-level education. The difference did not reach significance on the primary test (p = 0.066), although a higher proportion of females than males were in the highest educational category (75.7% vs 54.5%). The implications of this imbalance are examined in the sensitivity analysis below.

In Fig 1C (Question 4), both males and females exhibited a median income category representing the lowest income bracket (0–4,250 TL). No statistically significant gender-based income difference was detected (p = 0.089).

### Problem awareness and impact (Questions 5–6)

Fig 2 and Table 1 present problem awareness and its impact on participants.

In Fig 2A (Question 5), the most commonly reported factors across both genders were personal discomfort, feedback from others, and professional dental recommendation. No statistically significant gender difference was detected in the factors leading to problem awareness (p = 0.712; V = 0.16).

In Fig 2B (Question 6), the most commonly reported impact for both genders was appearance (males 40.9%, females 51.4%), followed by chewing and biting function (males 18.2%, females 29.7%). Further impacts were speech (males 18.2%, females 5.4%), temporomandibular joint problems and pain (males 6.8%, females 8.1%), and lack of self-confidence (males 6.8%, females 5.4%). Four male participants (9.1%) and no female participants reported not being affected by their condition; the breathing and sleep categories were not selected by any participant. No statistically significant gender difference was detected in how the condition affected participants (p = 0.170; V = 0.31).

### Functional evaluations: Eating and speech difficulties (Questions 7–9)

Fig 3 and Table 2 present functional evaluations related to eating and speech difficulties.

In Question 7, 48.6% of females reported frequent eating difficulties (Often/Always) compared with 27.3% of males. The difference was nominally significant (p = 0.032; r = 0.27, 95% CI 0.02 to 0.49) but did not remain significant after within-domain FDR correction (q = 0.085).

In Question 8, 59.5% of females reported frequently avoiding certain foods (Often/Always) compared with 31.8% of males. The difference was nominally significant (p = 0.043; r = 0.26, 95% CI 0.01 to 0.48) but did not remain significant after correction (q = 0.085).

In Question 9, 40.5% of females and 40.9% of males reported frequent speech difficulties (Often/Always). No statistically significant gender difference was detected (p = 0.864; r = 0.02, 95% CI −0.22 to 0.26).

### Functional evaluations: TMJ problems and breathing difficulties (Questions 10–12)

Fig 4 and Table 2 illustrate functional evaluations related to temporomandibular joint symptoms and breathing difficulties.

In Question 10, 27.0% of females reported frequent TMJ problems (Often/Always) compared with 18.2% of males, while 72.7% of males reported minimal such problems (Never/Rarely) versus 43.2% of females. Female participants reported more TMJ-related problems and pain than male participants at nominal significance (p = 0.013; r = 0.31, 95% CI 0.07 to 0.52), but the difference did not remain significant after correction (q = 0.076).

In Question 11, 27.0% of females and 20.5% of males reported frequent breathing difficulties in daily life (Often/Always), while 59.5% of females and 63.6% of males reported minimal difficulties (Never/Rarely). No statistically significant gender difference was detected (p = 0.614; r = 0.06, 95% CI −0.18 to 0.30).

In Question 12, 32.4% of females reported frequent sleep-related breathing difficulties (Often/Always) versus 20.5% of males, while 70.5% of males reported minimal such difficulties (Never/Rarely) compared with 48.6% of females. No statistically significant gender difference was detected (p = 0.178; r = 0.17, 95% CI −0.08 to 0.40).

### Psychosocial evaluations: Appearance concerns and self-confidence (Questions 13–16)

Fig 5 and Table 2 present psychosocial evaluations focused on appearance-related concerns and self-confidence.

In Question 13, 59.5% of females reported frequent appearance problems (Often/Always) compared with 50.0% of males. Only 8.1% of females reported minimal appearance concerns (Never/Rarely) versus 27.3% of males. No statistically significant difference was detected (p = 0.057; q = 0.133; r = 0.24, 95% CI −0.01 to 0.46).

In Question 14, 40.5% of females reported a frequent lack of social confidence (Often/Always) compared with 22.7% of males. In contrast, 50.0% of males reported minimal social confidence issues (Never/Rarely) versus 21.6% of females. Female participants experienced significantly greater lack of self-confidence when entering social environments (p = 0.006; q = 0.044; r = 0.35, 95% CI 0.10 to 0.55).

In Question 15, 62.2% of females reported a frequent lack of confidence when being photographed (Often/Always) compared with 43.2% of males, while 10.8% of females reported minimal concerns (Never/Rarely) versus 31.8% of males. Female participants reported greater lack of self-confidence when being photographed at nominal significance (p = 0.037; r = 0.26, 95% CI 0.02 to 0.48), but the difference did not remain significant after correction (q = 0.129).

In Question 16, 43.2% of females and 45.5% of males reported frequently hiding their teeth (Often/Always), while 35.1% of females and 43.2% of males reported minimal such behavior (Never/Rarely). No statistically significant difference was detected (p = 0.487; r = 0.09, 95% CI −0.16 to 0.32).

### Psychosocial evaluations: Social impact (Questions 17–19)

Fig 6 and Table 2 present responses to three social impact items.

In Question 17, 37.8% of females and 40.9% of males reported frequent worry about receiving negative comments (Often/Always), while 32.4% of females and 47.7% of males reported minimal worry (Never/Rarely). No statistically significant gender difference was detected (p = 0.425; r = 0.10, 95% CI −0.15 to 0.34).

In Question 18, 27.0% of females and 15.9% of males reported frequently receiving unpleasant comments (Often/Always), while 37.8% of females and 45.5% of males reported minimal such experiences (Never/Rarely). No statistically significant gender difference was detected (p = 0.347; r = 0.12, 95% CI −0.13 to 0.35).

In Question 19, 21.6% of females and 31.8% of males reported that appearance affects life success frequently (Often/Always), while 54.1% of females and 45.5% of males reported this rarely or never (Never/Rarely). No statistically significant difference was detected (p = 0.487; r = −0.09, 95% CI −0.32 to 0.16).

### Motivation for treatment (Question 20)

In Fig 7A and Table 3 (Question 20), the most commonly reported motivation source across all participants was personal motivation (“Myself”), endorsed by 62.2% of females and 47.7% of males. The second most frequent source was “My family and friends” (females 21.6%, males 25.0%), followed by “My orthodontist” (females 5.4%, males 20.5%) and “Patients who have previously had this treatment” (females 10.8%, males 6.8%). No participants in either gender group selected “My surgeon” as their primary motivation source. No statistically significant gender difference was detected in the distribution of motivation sources (p = 0.204; V = 0.24).

### Treatment expectations (Question 21)

In Fig 7B and Table 3 (Question 21), analysis revealed universally high expectations for improvement in external appearance (females 89.2%, males 88.6%) and smile (females 78.4%, males 59.1%). Approximately half of participants in both genders expected improvements in social life and self-confidence (females 51.4%, males 50.0%).

Female participants showed higher expectations for eating ability improvement (females 62.2%, males 45.5%) and TMJ problem resolution (females 43.2%, males 27.3%). Male participants demonstrated slightly higher expectations for speech improvement (males 54.5%, females 43.2%) and prevention of future dental problems (males 54.5%, females 48.6%).

Expectations for pain relief (females 24.3%, males 20.5%), ease in breathing (females 32.4%, males 27.3%), and comfortable sleep (females 35.1%, males 25.0%) were lower in both groups. No statistically significant gender differences were detected across the ten expectation categories (all p > 0.05). The confidence intervals for the differences in proportions were nevertheless wide; for example, the difference for smile was 19.3 percentage points (95% CI −1.1 to 37.2) and for eating ability 16.7 percentage points (95% CI −4.9 to 36.2) (Table 3).

### Pre-treatment information and satisfaction (Questions 22–24)

Fig 8 and Table 3 present evaluations of information provision and treatment satisfaction during the pre-operative phase.

In Question 22, 88.6% of males and 81.1% of females reported frequently receiving sufficient information from their orthodontist (Often/Always). The distributions differed across the full response range: the median response was “Always” among males and “Often” among females, and 65.9% of males selected “Always” compared with 32.4% of females. Male participants perceived significantly more adequate information provision from their orthodontists than female participants (p = 0.006; q = 0.018; r = −0.33, 95% CI −0.52 to −0.09).

In Question 23, 72.7% of males and 73.0% of females reported receiving sufficient information from their surgeon frequently (Often/Always). No statistically significant difference was detected (p = 0.157; r = −0.17, 95% CI −0.40 to 0.07).

In Question 24, 88.6% of males reported frequent satisfaction with pre-operative treatment (Often/Always) compared with 70.3% of females. Male participants experienced significantly higher satisfaction with their pre-operative treatment process than female participants (p = 0.013; q = 0.019; r = −0.30, 95% CI −0.50 to −0.06).

### Patient engagement and treatment recommendation (Questions 25–27)

Fig 9 and Table 3 illustrate patient engagement behaviors and treatment recommendation likelihood.

In Question 25, 54.5% of males and 51.4% of females reported having seen surgery videos or photos. No statistically significant gender difference was detected (p = 0.949; difference −3.2 percentage points, 95% CI −23.9 to 17.8). In Question 25.1, among those who had viewed these recordings (n = 43; 24 males, 19 females), no statistically significant difference in the reported effect was detected (p = 0.668); increased motivation was the most common response in both groups.

In Question 26, 25.0% of males and 32.4% of females reported having contacted individuals who previously underwent the treatment. No statistically significant gender difference was detected (p = 0.623; difference 7.4 percentage points, 95% CI −11.9 to 26.6). In Question 26.1, among those who had contacted previous patients (n = 23; 11 males, 12 females), no statistically significant difference in the reported effect was detected (p = 0.510); increased motivation was again the most common response.

In Question 27, 93.2% of males and 89.2% of females indicated they would recommend the treatment to others. No statistically significant gender difference was detected (p = 0.810; difference −4.0 percentage points, 95% CI −18.6 to 9.1).

### Sensitivity analyses

Two sensitivity analyses were performed. First, when the Benjamini-Hochberg procedure was applied across all 37 comparisons rather than within domains, no difference remained significant; the smallest adjusted values were q = 0.116 for Q14 and Q22 and q = 0.117 for Q10 and Q24. Full results, together with effect sizes for all comparisons, are given in S2 Table.

Second, because educational level was unevenly distributed between genders, ordinal logistic regression models were fitted for each of the seven nominally significant items with and without adjustment for education. Six of the seven associations were essentially unchanged after adjustment: Q7 (adjusted p = 0.045), Q10 (p = 0.012), Q14 (p = 0.005), Q15 (p = 0.048), Q22 (p = 0.004), and Q24 (p = 0.019). Food avoidance (Q8) was no longer significant after adjustment (p = 0.115), indicating that this association may be partly attributable to educational differences between the gender groups.

## Discussion

In this cross-sectional study of 81 adults with skeletal Class III malocclusion undergoing pre-surgical orthodontic preparation, item-level analysis of a 27-item questionnaire identified three gender differences that remained significant after within-domain false discovery rate correction: females reported greater lack of self-confidence in social settings, and males reported more adequate information provision from their orthodontist and higher satisfaction with the pre-operative orthodontic process. Four further differences, all in the direction of more frequent complaints among females (eating difficulty, food avoidance, temporomandibular joint (TMJ) pain, and lack of confidence when photographed), reached nominal significance only. No statistically significant differences were detected in problem awareness, speech, breathing, social impact, motivation, treatment expectations, or patient engagement, but the study was not powered to exclude small-to-moderate differences in these areas. The results are best regarded as exploratory and hypothesis-generating rather than definitive.

### Functional evaluations

Females reported more frequent eating difficulty (Q7, p = 0.032), food avoidance (Q8, p = 0.043), and TMJ problems and pain (Q10, p = 0.013), but none of these differences remained significant after within-domain correction (q = 0.076–0.085). No statistically significant differences were detected for speech (Q9, p = 0.864) or for breathing in daily life (Q11, p = 0.614) or during sleep (Q12, p = 0.178).

The direction of the eating-related findings is consistent with domain-level evidence. Bortoluzzi et al. reported significantly higher OQLQ Oral Function scores in Brazilian females [14], Johansson et al. reported the same pattern in a Swedish national register cohort [15], and Catt et al. found greater overall pre-treatment OQLQ concern among females in a four-centre UK study, although sub-domain statistics were not published [25]. Two studies diverge: Stagles et al. found no gender difference in the OQLQ Functional domain [12], and Williams et al. found none for self-reported eating difficulty [7]. Our TMJ finding accords with Rivera et al., who reported TMJ-related reasons for seeking surgery more frequently among females (33% vs 13%) [3].

The OQLQ Oral Function domain aggregates biting, chewing, food avoidance, eating in public, and jaw pain into a single score. If confirmed, an item-level pattern in which gender differences are concentrated in food avoidance and TMJ symptoms could partly account for the inconsistent domain-level results in the literature. This remains speculative: none of the three functional findings withstood correction for multiple testing, the food avoidance finding was additionally attenuated by adjustment for educational level, and the confidence intervals for speech and breathing do not exclude small-to-moderate differences.

### Psychosocial evaluations

Females reported significantly greater lack of self-confidence when entering social environments (Q14, p = 0.006, q = 0.044) and, at nominal significance only, when being photographed (Q15, p = 0.037, q = 0.129). No statistically significant differences were detected for appearance concerns (Q13, p = 0.057), hiding teeth when meeting people (Q16, p = 0.487), or the three social impact items (Q17–Q19, p = 0.347–0.487).

Q14 was the strongest psychosocial finding in the study and the only item in its domain to remain significant after correction. Stagles et al. reported a corresponding difference in the OQLQ Social domain [12], and Williams et al. found that females more often cited self-confidence improvement as a treatment motivation and more often felt embarrassed eating in public [7]. Bortoluzzi et al. and Johansson et al. reported non-significant social-domain differences, though both favoured the female-greater direction [14,15]. The OQLQ Social Aspects domain spans a broader set of constructs — worry about meeting people, perceived staring, and reactions to remarks — than the single construct assessed by Q14, which may explain the divergence.

The nominal Q15 finding is directionally consistent with Williams et al., who reported that females more often avoided smiling in photographs, and with the facial aesthetics domain differences reported by Bortoluzzi et al. and Johansson et al., which include items on being photographed or filmed [7,14,15]. Domain-level analyses cannot isolate this construct; the item-level approach does, although this finding did not survive correction and requires replication.

For Q16 (hiding teeth), Q17 (worry about negative comments), and Q18 (receiving unpleasant comments), the nearest comparators in Williams et al. reached significance: self-consciousness about tooth appearance, and having been teased about appearance [7]. The absence of a statistically significant difference here should not be read as a contradiction of that study. The confidence intervals for these items (Table 2) are compatible both with no difference and with small-to-moderate differences in the female-greater direction, and the constructs also differ: Williams et al. measured an internal feeling or a retrospective lifetime experience, whereas our items measured a current behaviour or perception at the time of survey completion. For Q19 (appearance affecting life success), no directly comparable published item was identified.

### Motivation and treatment expectations

No statistically significant gender difference was detected in the distribution of motivation sources (Q20, p = 0.204); personal motivation predominated in both groups (females 62.2%, males 47.7%). None of the ten treatment expectation categories differed significantly by gender (Q21.1–Q21.10, all p > 0.05), but several confidence intervals were wide, extending to differences of more than 30 percentage points for smile, eating ability, TMJ problems, and speech (Table 3), so moderate differences cannot be ruled out. Rivera et al. likewise found no gender differences in aesthetic or functional motivation categories [3]. Williams et al. reported that females more often cited self-confidence improvement and smile improvement, and males more often cited social life improvement [7]; these differences were not statistically significant in the present sample.

### Treatment process: Information and satisfaction

Males perceived significantly more adequate information provision from their orthodontist (Q22, p = 0.006, q = 0.018) and reported significantly higher satisfaction with the pre-operative orthodontic process (Q24, p = 0.013, q = 0.019), both differences being moderate in size (r = −0.33 and r = −0.30). No statistically significant difference was detected for perceived information from the surgeon (Q23, p = 0.157), despite identical item wording and response format, although the estimate was in the same direction (r = −0.17, 95% CI −0.40 to 0.07). The Q22 and Q24 differences survived within-domain correction and adjustment for educational level and, together with Q14, are the strongest exploratory findings in the study, although none survived correction across all 37 comparisons.

The absolute proportions reporting adequate information were high in both groups (88.6% of males and 81.1% of females), and the difference lies principally in the strength of endorsement rather than in whether information was perceived as adequate at all. Williams et al. found no gender differences in perceived information about braces or surgery, using a binary response format; the 5-point scale used here may be more sensitive to such graded differences [7]. The satisfaction finding is directionally consistent with the post-treatment literature: a systematic review by Pachêco-Pereira et al. identified sex as a significant determinant of satisfaction, with males more satisfied [26], a synthesis driven largely by Espeland et al.’s follow-up of 516 consecutively treated patients [27], and a more recent review by Almasri et al. reported that where a gender effect was found, females reported lower satisfaction [28]. Our finding suggests that such a difference may already be present during the pre-operative orthodontic phase.

Because the Q23 estimate was in the same direction as Q22 and the two confidence intervals overlap, the data do not establish that the difference in perceived information is specific to the orthodontist. The amount of contact patients had with the surgeon during pre-surgical orthodontic preparation was also not recorded (see Limitations).

### Patient engagement

No statistically significant gender differences were detected in any engagement item: exposure to surgery-related videos or photographs (Q25, p = 0.949), the perceived effect of such exposure among those reporting it (Q25.1, n = 43, p = 0.668), contact with previous patients (Q26, p = 0.623), the perceived effect of that contact (Q26.1, n = 23, p = 0.510), or willingness to recommend the treatment (Q27, p = 0.810). The conditional items were answered by small subgroups and have very limited power, and the confidence intervals for the binary engagement items extended to differences of approximately 19–27 percentage points, so these results should not be taken as evidence that engagement is similar in both genders. Across the whole sample, exposure to visual material and contact with previous patients most commonly increased motivation for surgery, consistent with Williams et al., who reported that most patients shown photographs of previous cases or given the opportunity to meet a former patient found this helpful [7], and with AlKharafi et al., who reported a comparably high recommendation rate in a Kuwaiti cohort [29].

### Methodological considerations

Two features of the analysis bear on how these findings should be read. First, 37 comparisons were performed. Inference was based on within-domain false discovery rate-adjusted values, under which Q14, Q22, and Q24 remained significant, whereas Q7, Q8, Q10, and Q15 did not; these four nominal findings require independent replication. Within-domain correction is less stringent than correction across the whole questionnaire, and when the procedure was applied across all 37 comparisons no difference remained significant (smallest q = 0.116). Even the three strongest exploratory findings should therefore be regarded as hypotheses for confirmation rather than established differences.

Second, the a priori sample size calculation was specified as a one-sample test and did not address the between-gender comparisons. For the realized group sizes, the study had 37% power to detect the originally assumed effect (d = 0.37) and 80% power only for effects of d ≈ 0.63 or larger. Non-significant findings for problem awareness, speech, breathing, social impact, motivation, expectations, and engagement are therefore compatible with no gender difference, but equally compatible with small-to-moderate differences that this sample could not detect, as the confidence intervals in Tables 1–3 show. They should not be interpreted as evidence of equivalence. A confirmatory study powered to detect d = 0.37 with 80% power would require approximately 116 participants per group.

### Limitations and future directions

Several limitations should be considered when interpreting these findings.

First, the questionnaire was assembled by the investigators from multiple published sources and was not subjected to formal cross-cultural adaptation, test-retest reliability testing, or construct validity assessment. Although its constructs overlap with those of the OQLQ, the response format differs, and the psychometric properties established for validated instruments do not transfer to it. Internal consistency in the present sample was acceptable across all three Likert domains, but the instrument should be regarded as investigator-developed.

Second, the number of comparisons performed and the sample size available constrain the strength of the inferences that can be drawn, as set out in the preceding section.

Third, the number and timing of patient–surgeon interactions during the pre-surgical orthodontic phase were not systematically recorded. In our clinical care model, patients were evaluated jointly by the orthodontist and surgeon when the decision for orthognathic surgery was made, with further joint consultations occurring during the pre-surgical orthodontic preparation period and again closer to surgery. However, individual consultation frequency could not be reconstructed retrospectively. Therefore, the Q22 and Q23 findings, and the absence of any participant identifying the surgeon as a primary motivation source (Q20), should be interpreted with this uncertainty in mind.

Fourth, the analyzed sample is by design a complete-case sample: four questionnaires were excluded because they were incomplete or incorrectly completed, and it is not known whether those patients differed systematically from those included. The number of patients declining to participate was zero, but eligibility and exclusion counts were reconstructed retrospectively rather than recorded prospectively.

Fifth, educational level was unevenly distributed between the gender groups. Adjustment for education left six of seven findings essentially unchanged, but attenuated the food avoidance association, and residual confounding by unmeasured socioeconomic factors cannot be excluded.

Sixth, the single-centre, cross-sectional design restricts generalizability. All participants were recruited from one university clinic in Istanbul, and cultural, socioeconomic, and healthcare-system factors specific to this setting may have shaped the responses. The design also captures a single time point and cannot address how gender differences evolve across the treatment pathway; Catt et al. reported that gender differences in social quality of life had disappeared two years after surgery [25].

Seventh, self-reported data are subject to social desirability bias and to individual variation in the interpretation of Likert anchors.

Eighth, the study included only adults with skeletal Class III malocclusion, and the findings may not generalize to Class II or other dentofacial deformities.

Future research should prioritize multi-centre longitudinal studies with larger samples and formally validated instruments, including patients with other skeletal malocclusion classes and cross-cultural comparisons. Interventional studies testing whether tailored communication strategies reduce gender differences in perceived information adequacy and treatment satisfaction would be of particular clinical value.

## Conclusion

In this exploratory, single-centre, cross-sectional study of 81 adults with skeletal Class III malocclusion, item-level analysis identified seven gender differences at the unadjusted threshold, of which three remained significant after within-domain correction for multiple comparisons and after adjustment for educational level: females reported greater lack of social self-confidence, while males reported more adequate information provision from their orthodontist and higher satisfaction with the pre-operative orthodontic process. The other four, nominal differences in eating- and TMJ-related complaints and photographic self-confidence, did not withstand correction. No statistically significant differences were detected in problem awareness, treatment motivation, treatment expectations, or patient engagement behaviours, but because the study was not powered a priori for gender comparisons, these results do not establish that the genders are similar in these respects.

These findings suggest that the pre-surgical orthodontic phase merits closer attention as a point in the treatment pathway where patient experience may differ by gender. Given the exploratory design, the number of comparisons performed, and the single-centre sample, they require confirmation in multi-centre, longitudinal, and cross-cultural studies before informing changes to clinical practice.

## Supporting information

S1 Fig Participant recruitment and inclusion flow diagram. (TIF)

S1 Table Full 27-item questionnaire administered to participants. (DOCX)

S2 Table Unadjusted p-values, Benjamini-Hochberg false discovery rate-adjusted values within domains and across all 37 comparisons, and effect sizes for all comparisons, with 95% confidence intervals where applicable. (XLSX)

S1 Dataset De-identified item-level responses. (XLSX)

S1 File STROBE checklist for cross-sectional studies. (DOCX)
