# Supplementary material for: Gender differences in pre-surgical motivations, expectations, and patient-reported outcomes in Class III orthognathic surgery patients: An item-level analysis
Source: PLoS One. 2026 Nov 5;21(11):e0362253. doi: 10.1371/journal.pone.0362253 (PMC13739105; doi:10.1371/journal.pone.0362253)
Supplement: Supplementary file 1 [file pone.0362253.s002.docx]

GENDER DIFFERENCES IN PRE-SURGICAL MOTIVATIONS, EXPECTATIONS, AND PATIENT-REPORTED OUTCOMES IN CLASS III ORTHOGNATHIC SURGERY PATIENTS: AN ITEM-LEVEL ANALYSIS

# DEMOGRAPHIC INFORMATION

**1. Gender:**

(1) Female

(2) Male

**2. Age:**

**3. Educational Level:**

(1) Primary school

(2) High school

(3) University

(4) Graduate degree

**4. Monthly net income:**

(1) 0 - 4,250 TL

(2) 4,250 TL - 7,500 TL

(3) 7,500 TL - 12,000 TL

(4) Over 12,000 TL

# PROBLEM AWARENESS

**5. What caused you to notice these problems?**

(1) I was feeling uncomfortable myself

(2) People around me told me

(3) My family was disturbed by it

(4) My dentist said I might need orthodontic treatment

(5) My orthodontist informed me

**6. How does your current condition mainly affect you? (Please select only one option)**

(1) Chewing and biting

(2) External appearance and aesthetics

(3) Speech

(4) Temporomandibular joint (TMJ) and pain problems

(5) Breathing difficulties

(6) Sleep problems

(7) Lack of self-confidence

(8) I'm not affected

# FUNCTIONAL EVALUATIONS

**7. Do you have difficulty eating?**

(1) Never

(2) Rarely

(3) Sometimes

(4) Often

(5) Always

**8. Do you avoid eating certain foods due to problems with your teeth and jaw?**

(1) Never

(2) Rarely

(3) Sometimes

(4) Often

(5) Always

**9. Do you have difficulty speaking and pronouncing words?**

(1) Never

(2) Rarely

(3) Sometimes

(4) Often

(5) Always

**10. Does your current condition cause temporomandibular joint problems and pain?**

(1) Never

(2) Rarely

(3) Sometimes

(4) Often

(5) Always

**11. Do you have difficulty breathing in daily life?**

(1) Never

(2) Rarely

(3) Sometimes

(4) Often

(5) Always

**12. Do you have difficulty breathing while sleeping?**

(1) Never

(2) Rarely

(3) Sometimes

(4) Often

(5) Always

# PSYCHOSOCIAL EVALUATIONS

**13. Do you have problems with your external appearance?**

(1) Never

(2) Rarely

(3) Sometimes

(4) Often

(5) Always

**14. Do you feel lack of self-confidence when entering any social environment?**

(1) Never

(2) Rarely

(3) Sometimes

(4) Often

(5) Always

**15. Do you feel lack of self-confidence when taking photos?**

(1) Never

(2) Rarely

(3) Sometimes

(4) Often

(5) Always

**16. Do you hide your teeth when you meet people?**

(1) Never

(2) Rarely

(3) Sometimes

(4) Often

(5) Always

**17. Are you worried about receiving negative comments about your appearance?**

(1) Never

(2) Rarely

(3) Sometimes

(4) Often

(5) Always

**18. Have you ever received unpleasant comments about your appearance?**

(1) Never

(2) Rarely

(3) Sometimes

(4) Often

(5) Always

**19. Do you think your appearance affects your life success?**

(1) Never

(2) Rarely

(3) Sometimes

(4) Often

(5) Always

# MOTIVATION AND EXPECTATIONS

**20. What is your main source of motivation for this treatment?**

(1) Myself

(2) My family and friends

(3) My orthodontist

(4) My surgeon

(5) Patients who have previously had this treatment

**21. What are your expectations as a result of this treatment? (You may select multiple answers)**

(1) Improvement in my external appearance

(2) Improvement in my smile

(3) Improvement in my social life and self-confidence

(4) Improvement in my eating ability

(5) Improvement in my speech

(6) Pain relief

(7) Resolution of temporomandibular joint (TMJ) problems

(8) Ease in breathing

(9) Comfortable sleep

(10) Prevention of future problems with my teeth

# TREATMENT PROCESS EVALUATION

**22. Have you received sufficient information about the treatment from your orthodontist?**

(1) Never

(2) Rarely

(3) Sometimes

(4) Often

(5) Always

**23. Have you received sufficient information about the treatment from your surgeon?**

(1) Never

(2) Rarely

(3) Sometimes

(4) Often

(5) Always

**24. Are you satisfied with your pre-operative orthodontic treatment process?**

(1) Never

(2) Rarely

(3) Sometimes

(4) Often

(5) Always

# PATIENT ENGAGEMENT

**25. Have you seen videos and photos related to before and after surgery?**

(1) Yes

(2) No

*If yes, answer question 25.1. If no, continue with question 26.*

**25.1. How did these recordings affect you?**

(1) My motivation increased

(2) My fears and worries increased

(3) I was not affected

**26. Have you contacted patients who have previously had surgery?**

(1) Yes

(2) No

*If yes, answer question 26.1. If no, continue with question 27.*

**26.1. How did this contact affect you?**

(1) My motivation increased

(2) My fears and worries increased

(3) I was not affected

**27. Would you recommend this treatment to other patients in your condition?**

(1) Yes

(2) No

(3) I'm undecided

**Note:** This questionnaire was adapted from Williams et al., Rivera et al., Türker et al., and Al-Rawee et al., and incorporates elements from the Orthognathic Quality of Life Questionnaire (OQLQ).
