# Supplementary material for: Gender differences in pre-surgical motivations, expectations, and patient-reported outcomes in Class III orthognathic surgery patients: An item-level analysis
Source: PLoS One. 2026 Nov 5;21(11):e0362253. doi: 10.1371/journal.pone.0362253 (PMC13739105; doi:10.1371/journal.pone.0362253)
Supplement: Supplementary file 2 [file pone.0362253.s005.docx]

**STROBE Statement—Checklist of items for reports of cross-sectional studies**

***Gender differences in pre-surgical motivations, expectations, and patient-reported outcomes in Class III orthognathic surgery patients: an item-level analysis***

| **Section/Topic** | **Item No** | **Recommendation** | **Reported** |
| --- | --- | --- | --- |
| **Title and abstract** | | | |
| **Title and abstract** | 1(a) | Indicate the study’s design with a commonly used term in the title or the abstract | Title: “… an item-level analysis”; Abstract, Methods: “single-center, cross-sectional study” |
|  | 1(b) | Provide in the abstract an informative and balanced summary of what was done and what was found | Abstract (Objective, Methods, Results, Conclusions) |
| **Introduction** | | | |
| **Background/ rationale** | 2 | Explain the scientific background and rationale for the investigation being reported | Introduction, paragraphs 1–4 |
| **Objectives** | 3 | State specific objectives, including any prespecified hypotheses | Introduction, final paragraph: “The aim of this study was to evaluate … with particular attention to gender differences at the item level” |
| **Methods** | | | |
| **Study design** | 4 | Present key elements of study design early in the paper | Materials and Methods, Study Design and Setting: “single-center, cross-sectional study” |
| **Setting** | 5 | Describe the setting, locations, and relevant dates | Materials and Methods, Study Design and Setting: Department of Orthodontics, Faculty of Dentistry, Istanbul University; ethics approval decision no. 587, meeting no. 119, protocol no. 2022/16, dated 8 March 2022; recruitment 1 April – 1 October 2022 |
| **Participants** | 6(a) | Give the eligibility criteria, and the sources and methods of selection of participants | Participants section: inclusion (age ≥18 years, skeletal Class III, pre-surgical orthodontic preparation) and exclusion criteria (cleft lip and/or palate, congenital deformities affecting dentofacial development, prior orthodontic or orthognathic treatment); restriction of enrollment to Class III explained; participant flow in S1 Fig |
| **Variables** | 7 | Clearly define all outcomes, exposures, predictors, potential confounders, and effect modifiers | Survey Instrument section: 27-item questionnaire across 7 domains; gender as the grouping variable; full instrument in S1 Table |
| **Data sources/ measurement** | 8* | For each variable of interest, give sources of data and details of methods of assessment | Survey Instrument: 5-point frequency Likert scales, single- and multiple-choice items; adaptation process described (rewording for the Turkish setting, conversion of binary or bother-based responses to a 5-point frequency format, investigator-written items); sources cited [3,7,16,17] and OQLQ [18,19]; questionnaires self-completed in the clinic; full instrument in S1 Table |
| **Bias** | 9 | Describe any efforts to address potential sources of bias | Limitations: self-report bias, complete-case sample, retrospective reconstruction of eligibility counts, unmeasured socioeconomic confounding; Wilson Score confidence intervals for all proportions; education-adjusted sensitivity analysis reported in Results |
| **Study size** | 10 | Explain how the study size was arrived at | Sample Size Determination: a priori power analysis performed February 2022 and documented in the approved study protocol — one-sample t-test, d = 0.37, α = 0.05 (one-tailed), power = 0.95, required total n = 81 (actual power 0.951). This calculation did not address the between-gender comparisons, which are therefore presented as exploratory; for the realized groups (37 vs 44) power for d = 0.37 was 0.37 and the minimum detectable d with 80% power ≈ 0.63 |
| **Quantitative variables** | 11 | Explain how quantitative variables were handled in the analyses | Statistical Analysis: continuous and ordinal variables by Mann-Whitney U; nominal variables by chi-square, with Fisher’s or Monte Carlo exact tests where expected cell counts were below 5 |
| **Statistical methods** | 12(a) | Describe all statistical methods, including those used to control for confounding | Statistical Analysis: Mann-Whitney U, chi-square, Fisher’s and Monte Carlo exact tests; effect sizes for all comparisons, with 95% CIs where applicable (rank-biserial r with Cliff’s CI, differences in proportions with Newcombe CI, Cramér’s V without CI); Wilson Score confidence intervals; Cronbach’s alpha with bootstrap confidence intervals; Benjamini-Hochberg FDR adjustment within domains (Significance Threshold and Multiplicity); ordinal logistic regression adjusted for educational level (Results, Sensitivity analyses) |
|  | 12(b) | Describe any methods used to examine subgroups and interactions | Gender as the primary grouping variable; conditional analyses for Q25.1 (n = 43) and Q26.1 (n = 23); within-domain analyses reported in S2 Table |
|  | 12(c) | Explain how missing data were addressed | No missing data for any analysed variable. Four incomplete or incorrectly completed questionnaires were excluded before analysis in accordance with the study protocol (S1 Fig); the resulting complete-case nature of the sample is acknowledged in Limitations |
|  | 12(d) | If applicable, describe analytical methods taking account of sampling strategy | Not applicable (consecutive eligible patients at a single center; recruitment stopped once the pre-specified target sample was reached) |
|  | 12(e) | Describe any sensitivity analyses | Results, Sensitivity analyses: Benjamini-Hochberg correction applied across all 37 comparisons (S2 Table); ordinal logistic regression with and without adjustment for educational level. Multiplicity and its consequences are described in Methods (Significance Threshold and Multiplicity) and Discussion (Methodological considerations) |
| **Results** | | | |
| **Participants** | 13*(a) | Report numbers of individuals at each stage of study | Participants section and S1 Fig: 105 assessed for eligibility, 20 not enrolled, 85 enrolled and completed the questionnaire, 4 excluded, 81 analysed (37 females, 44 males) |
|  | 13*(b) | Give reasons for non-participation at each stage | No eligible patient declined to participate. Reasons for non-enrollment are given in S1 Fig: cleft lip and/or palate (n = 10), other syndromic craniofacial conditions (n = 2), skeletal Class II malocclusion following the diagnostic restriction (n = 8) |
|  | 13*(c) | Consider use of a flow diagram | S1 Fig. Participant recruitment and inclusion flow diagram |
| **Descriptive data** | 14*(a) | Give characteristics of study participants | Table 1 and Fig 1: age, educational level and monthly income by gender; Cronbach’s alpha for the three Likert domains reported in Baseline Characteristics |
|  | 14*(b) | Indicate number of participants with missing data for each variable of interest | No missing data for any analysed variable; conditional items (Q25.1, Q26.1) report their subsample sizes |
| **Outcome data** | 15* | Report numbers of outcome events or summary measures | Tables 1–3: p-values and the test used for every item; Results text: percentages for each item, reported consistently as the combined Often/Always categories |
| **Main results** | 16(a) | Give unadjusted estimates and, if applicable, confounder-adjusted estimates and their precision | Results and Tables 1–3: unadjusted p, within-domain FDR-adjusted q, and effect sizes for every comparison, with 95% confidence intervals where applicable; 95% Wilson Score confidence intervals for proportions; Figs 1–9; education-adjusted p-values in Results, Sensitivity analyses |
|  | 16(b) | Report category boundaries when continuous variables were categorized | Age reported as continuous; educational level and income as ordinal categories with boundaries given in the S1 Dataset codebook (Table 1, Fig 1) |
|  | 16(c) | If relevant, consider translating estimates of relative risk into absolute risk | Not applicable |
| **Other analyses** | 17 | Report other analyses done—eg analyses of subgroups and interactions, and sensitivity analyses | Results, Sensitivity analyses: Benjamini-Hochberg correction across all 37 comparisons (S2 Table) and education-adjusted ordinal logistic regression; conditional analyses for Q25.1 (n = 43) and Q26.1 (n = 23) |
| **Discussion** | | | |
| **Key results** | 18 | Summarise key results with reference to study objectives | Discussion, opening paragraph: three gender differences remaining significant after within-domain FDR correction, four nominal differences, and non-significant domains interpreted with reference to confidence intervals and power |
| **Limitations** | 19 | Discuss limitations of the study, taking into account sources of potential bias or imprecision | Limitations and future directions: eight numbered limitations — investigator-developed instrument without formal validation, multiplicity and sample size, undocumented patient–surgeon contact, complete-case sample and retrospectively reconstructed counts, educational imbalance, single-centre cross-sectional design, self-report bias, and restriction to Class III |
| **Interpretation** | 20 | Give a cautious overall interpretation of results | Discussion subsections and Methodological considerations; Conclusion: findings described as exploratory and hypothesis-generating, requiring confirmation before informing clinical practice |
| **Generalisability** | 21 | Discuss the generalisability (external validity) of the study results | Limitations: single-centre Istanbul setting; multi-centre, longitudinal and cross-cultural replication recommended |
| **Other information** | | | |
| **Funding** | 22 | Give the source of funding and the role of the funders | Financial disclosure provided in the online submission form; the study received no external funding. |
